# Combinations of Single-Gene Biomarkers Can Precisely Stratify 1,028 Adult Gliomas for Prognostication

**DOI:** 10.3389/fonc.2022.839302

**Published:** 2022-04-26

**Authors:** Aden Ka-Yin Chan, Zhi-Feng Shi, Kay Ka-Wai Li, Wei-Wei Wang, Hong Chen, Nellie Yuk-Fei Chung, Danny Tat-Ming Chan, Wai-Sang Poon, Herbert Ho-fung Loong, Xian-Zhi Liu, Zhen-Yu Zhang, Ying Mao, Ho-Keung Ng

**Affiliations:** ^1^ Department of Anatomical and Cellular Pathology, The Chinese University of Hong Kong, Shatin, Hong Kong SAR, China; ^2^ Hong Kong and Shanghai Brain Consortium (HSBC), Hong Kong, Hong Kong SAR, China; ^3^ Department of Neurosurgery, Huashan Hospital, Fudan University, Shanghai, China; ^4^ Department of Pathology, The First Affiliated Hospital of Zhengzhou University, Zhengzhou, China; ^5^ Department of Pathology, Huashan Hospital, Fudan University, Shanghai, China; ^6^ Division of Neurosurgery, Department of Surgery, The Chinese University of Hong Kong, Shatin, Hong Kong SAR, China; ^7^ Department of Clinical Oncology, The Chinese University of Hong Kong, Shatin, Hong Kong SAR, China; ^8^ Department of Neurosurgery, The First Affiliated Hospital of Zhengzhou University, Zhengzhou, China

**Keywords:** gliomas, IDH1/2, 1p19q, TERT, BRAF, EGFR, 10q, H3

## Abstract

Advanced genomic techniques have now been incorporated into diagnostic practice in neuro-oncology in the literature. However, these assays are expensive and time-consuming and demand bioinformatics expertise for data interpretation. In contrast, single-gene tests can be run much more cheaply, with a short turnaround time, and are available in general pathology laboratories. The objective of this study was to establish a molecular grading scheme for adult gliomas using combinations of commonly available single-gene tests. We retrospectively evaluated molecular diagnostic data of 1,275 cases of adult diffuse gliomas from three institutions where we were testing for IDH1/2 mutation, TERTp mutation, 1p19q codeletion, EGFR amplification, 10q deletion, BRAF V600E, and H3 mutations liberally in our regular diagnostic workup. We found that a molecular grading scheme of Group 1 (1p19q codeleted, IDH mutant), Group 2 (IDH mutant, 1p19q non-deleted, TERT mutant), Group 3 (IDH mutant, 1p19q non-deleted, TERT wild type), Group 4 (IDH wild type, BRAF mutant), Group 5 (IDH wild type, BRAF wild type and not possessing the criteria of Group 6), and Group 6 (IDH wild type, and any one of TERT mutant, EGFR amplification, 10q deletion, or H3 mutant) could significantly stratify this large cohort of gliomas for risk. A total of 1,028 (80.6%) cases were thus classifiable with sufficient molecular data. There were 270 cases of molecular Group 1, 59 cases of molecular Group 2, 248 cases of molecular Group 3, 27 cases of molecular Group 4, 117 cases of molecular Group 5, and 307 cases of molecular Group 6. The molecular groups were independent prognosticators by multivariate analyses and in specific instances, superseded conventional histological grades. We were also able to validate the usefulness of the Groups with a cohort retrieved from The Cancer Genome Atlas (TCGA) where similar molecular tests were liberally available. We conclude that a single-gene molecular stratification system, useful for fine prognostication, is feasible and can be adopted by a general pathology laboratory.

## Introduction

There has been a vast increase in our knowledge of the molecular pathology of central nervous system (CNS) tumors, and a new WHO classification will soon be published (1). The WHO 2016 Classification already introduced for the first time molecular criteria in the classification of gliomas by IDH status and the molecular groups of medulloblastomas (2). In many advanced centers, brain tumor samples are now routinely tested with genomic techniques, such as targeted sequencing, RNAseq, and genome-wide DNA methylation profiling (3–6). The latter has become a regular genomic diagnostic tool in many centers (7–9), and many tumor entities in the upcoming WHO classification will have the methylation groups listed as desirable diagnostic criteria (1). However, genomic tests are still expensive, especially in countries with a national health system, and also typically, their results only come back several weeks later. Interpretation of genomic data also requires expertise and reporting of genomic tests, especially those conducted by commercial companies, is variable. In contrast, single-gene tests are faster for results, often done in-house, and are much cheaper. Results of many single-gene tests, e.g., 1p19q, IDH genotyping, are needed soon after the surgical resection so that planning for further treatment can be made and patients can receive appropriate counseling as soon as possible after the operation. There is therefore a need for precise molecular diagnosis and grading of brain tumors using single-gene tests.

Isocitrate dehydrogenase (IDH) genotype has been shown to be a major classifier and prognosticator for gliomas (10, 11) and WHO 2016 classifies astrocytic tumors as to whether they are IDH mutant or IDH wild type. Together with another group, we were the first to show the usefulness of mutations of TERT promoter (TERTp), when combined with IDH genotype, in the precise prognostication of low-grade gliomas (12, 13). The Cancer Genome Atlas (TCGA) showed that a combination of IDH genotype and 1p19q status stratified survivals well for the low-grade gliomas (14), but Eckel-Passow et al. in the same issue of *New England Journal of Medicine*, in addition to using IDH and 1p19q, introduced also TERT mutation to stratify low-grade gliomas (15). BRAF is also a gene that can be easily tested in most laboratories without too much difficulty with a mutation hotspot at V600E. We showed that in the younger adults, it is associated with good survival, and similarly, good survival with BRAF V600E mutated gliomas was shown in smaller series (16–18). Phillips et al. showed that among BRAF mutated anaplastic pleomorphic xanthoastrocytoma, the regular molecular hallmarks of glioblastomas, e.g., EGFR amplification, 10q loss, were uncommon (19). Mutations of the chromatin genes, H3.3 and H3.1, were also found to be poor prognosticators for gliomas, and the poor prognosis is not limited to tumors located in mid-line, nor were mutations restricted to pediatric high-grade gliomas (20–24).

We hypothesized that combinations of genes IDH1/2, 1p19q, TERTp, EGFR, BRAF, TERTp, 10q, and H3 status can stratify gliomas for risk and may even be superior to conventional histological grading for prognostication. We recognize that this gene list is not exhaustive as regards the vast amount of literature concerning the molecular pathology of gliomas. However, since there is a limit as to how many single-gene tests an in-house facility can possibly carry out with a reasonable turnover time, we hypothesize that such a list would adequately molecularly grade a glioma and that it could be superior to conventional histological grading in some instances.

## Materials and Methods

### Patients

A total of 1,275 adult diffuse gliomas diagnosed as astrocytic or oligodendroglial from patients aged 18 years or above were unselectively and retrospectively retrieved from the pathology archives of three institutions: Prince of Wales Hospital, Chinese University of Hong Kong, Huashan Hospital, Fudan University, Shanghai and First Affiliated Hospital of Zhengzhou University, Zhengzhou. Pediatric gliomas were excluded due to the vastly different molecular pathology between adult and pediatric gliomas (25, 26). Some cases were part of published studies (27–29), and the others were diagnosed between 2011 and 2017, and in the latter cases, molecular tests were done as part of the diagnostic workup. Cases lacking enough clinical information or survival data were excluded. Histological sections were centrally reviewed by two experienced neuropathologists before the present analyses (H-KN and HC). Histological grading from Grade II to Grade IV was made also according to the WHO 2016 Classification. The diagnostic criteria for Grade III tumors were increased mitotic activity, nuclear atypia, and high cellularity as described in WHO 2016, and the defining criteria for Grade IV tumors (glioblastomas) were either the presence of necrosis or endothelial proliferation. Ethics approvals were obtained from The Joint Chinese University of Hong Kong–New Territories East Cluster Clinical Research Ethics Committee; the Ethics Committees of Huashan Hospital, Shanghai; and The First Affiliated Hospital of Zhengzhou University, Zhengzhou. The study was performed in accordance with the Declaration of Helsinki.

Demographics and survival data were obtained from the hospital information systems of the three hospitals. The protocol for treatment for adult gliomas of Grades II–IV in all three hospitals was maximal safe resection in the first instance. Some but not all patients received adjuvant radio-chemotherapy due to individual clinical situations (**Table 1**), and this information was obtained from the hospital information systems. Overall survival (OS) was obtained either with the hospital information systems or by phone calls as practiced in our previous studies (30–32). OS was defined as the period of time between operation and death or the last follow-up.

**Table 1:** Clinical characteristics of 1,275 diffuse gliomas.

	All cases	Molecular group I	Molecular group 2	Molecular group 3	Molecular group 4	Molecular group 5	Molecular group 6
	(N = 1275)	(N = 270)	(N = 59)	(N = 248)	(N = 27)	(n = 117)	(N = 307)
Age (Mean/Median/Range)	45.6 *I* 45 *I* 19-82	44.2 *I* 43 *I* 23-71	44 *I* 46 *I* 22-67	41.2 *I* 40 *I* 22-79	37.7 *I* 33 *I* 19-71	41.2 *I* 40 *I* 19-75	52.3 *I* 56 *I* 19-82
Gender							
Male	746 (58.5%)	161 (59.6%)	36 (61%)	145 (58.5%)	12 (44.1%)	72 (61.5%)	177 (57.7%)
Female	529 (41.5%)	109 (40.4%)	23 (39%)	103 (41.5%)	15 (55.6%)	45 (38.5%)	130 (42.3%)
H istologic grade							
Grade II	554 (43.5%)	206 (76.3%)	43 (72.9%)	164 (66.1%)	5 (18.5%)	33 (28.2%)	41 (13.4%)
Grade III	304 (23.8%)	64 (23.7%)	15 (25.4%)	67 (27%)	4 (14.8%)	34 (29.1%)	60 (19.5%)
GradeIV	417 (32.7%)	0	I (1.7%)	17 (6.9%)	18 (66.7%)	50 (42.7%)	206 (67.1%)
Operation							
Total resection	828 (64.9%)	291 (81.1%)	40 (67.8%)	163 (65.7%)	16 (59.3%)	51 (43.6%)	174 (56.7%)
Non-total resection	386 (30.3%)	51 (18.9%)	18 (30.5%)	71 (28.6%)	4 (14.8%)	52 (44.4%)	114 (37.1%)
Not available	61 (4.8%)	0	I (1.7%)	14 (5.6%)	7 (25.9%)	14 (12%)	19 (6.2%)
Radiotherapy							
Yes	692 (54.3%)	203 (75.2%)	43 (72.9%)	185 (74.6%)	16 (59.3%)	75 (64.1%)	124 (40.4%)
No	202 (15.8%)	45 {16.7%)	13 (22%)	45 (18.1%)	4 (14.8%)	26 (22.2%)	46 (15%)
Not available	381 (29.9%)	22 (8.1%)	3 (5.1%)	18 (7.3%)	7 (25.9%)	16 (13.7%)	137 (44.6%)
Chemotherapy							
Yes	622 (48.8%)	176 (65.2%)	34 (57.6%)	161 (64.9%)	17 (63%)	70 (59.8%)	108 (35.2%)
No	290 (22.7%)	72 (26.7%)	22 (37.3)	69 (27.8%)	3 (I 1.1%)	32 (27.4%)	62 (20.2%)
Not available	363 (28.5%)	22 (8.1%)	3 (5.1%)	18 (7.3%)	7 (25.9%)	15 {12.8%)	137 (44.6%)
Overall survival (Mean/Median) (years)	6.7 *I* 3.9	12.3 *I* 13.3	9.6 111.3	7.6 16.1	6. 7 *I* 5.1	5. 0/1.8	1.8 *I* 1.0

Results of molecular tests for this cohort were all retrieved. We had a long-term interest in TERTp mutations in adult gliomas (12, 27–29), and therefore adult gliomas diagnosed between the years 2011 and 2017 at our three institutions had been examined extensively by this test. Cases diagnosed prior to this period of time were part of the previous studies (27–29). EGFR amplification, H3.3 and H3.1 mutations, and 10q deletion were also examined as much as tissues would allow in IDH wild-type gliomas because of our previous findings of their prognosticative value and also findings from the literature (14, 33, 34). BRAF V600E mutations were also evaluated liberally whenever tissue was available during this period because of our previous interest in BRAF (18) and also the possibility of target therapy (35). At our three institutes, IDH status was routinely obtained by Sanger sequencing, and all IDH mutant gliomas were evaluated for 1p19q codeletion; some IDH wild-type gliomas also had 1p19q analysis because the two tests were sometimes requested simultaneously. Fluorescence *in situ* hybridization (FISH) for CDKN2A homozygous deletion was also evaluated in some cases as previously described (32). The methodology used for the single-gene tests was the same for the three institutions.

### Mutation Analysis

The hotspots for IDH1/2, TERTp, BRAF, H3.3, and H3.1 mutations were evaluated by Sanger sequencing as described (18, 30). In brief, tissue sections obtained from either macrodissection or direct scrapping off the slides were placed into Tris–HCl buffer (pH 8.5) containing proteinase K. The mixture was then incubated at 56°C for overnight followed by 98°C for 10 min. The crude lysate was then mixed with primers, KAPA Robust HotStart ReadyMix (Sigma, St. Louis, MO, USA)/KAPA HiFi HotStart ReadyMix (Sigma), for PCR amplification. PCR products were visualized on an electrophoresis gel, cleaned with a spin column-based PCR product purification kit, and sequenced with BigDye Terminator Cycle Sequencing kit v1.1 (Life Technologies, Carlsbad, CA, USA). The PCR primers can be found in **Supplementary Table 1**.

### Fluorescence *In Situ* Hybridization Analysis

EGFR amplification, 10q deletion, 1p19q codeletion, and CDKN2A homozygous deletion were evaluated by FISH. The BAC clone (CTD-2199A14) containing the genomic sequences of the 7p11.2 and a centromere probe (CEP7, Vysis, Downers Grove, IL, USA) was used for EGFR amplification detection. Vysis LSI PTEN/CEP 10 FISH Probe Kit was applied for detection of 10q loss. Vysis 1p36/1q25 and 19q13/19p13 FISH Probe Kit (Vysis) was used to examine 1p19q codeletion. Vysis LSI CDKN2A SpectrumOrange/CEP 9 SpectrumGreen Probes (Vysis) was employed to study CDKN2A. At least 100 non-overlapping signals were counted and analyzed in each case. EGFR amplifications were considered when >5% recorded cells displayed many tight clusters or a ratio of the target (red) to reference (green) signal >2 (33, 36). Deletion for 1p, 19q, and 10q was considered when more than 25% of counted nuclei presented one target (orange) signal and two references (green) signals (37). CDKN2A homozygous deletion was considered when >20% of tumor cells showed loss of two signals, in the presence of two reference signals (32).

### The Cancer Genome Atlas Data Retrieval

A cohort with a similar gene set from TCGA was also retrieved (10, 14). Grades II–IV gliomas aged 18 or above were selected, and the clinical information and relevant molecular data concerning IDH1/2 mutations, 1p19q codeletion, BRAF V600E, 10q deletion, EGFR amplification, and H3 mutations were downloaded. They were then stratified for survival according to our molecular grading scheme.

### Statistical Analysis

Statistical analysis was conducted on IBM SPSS software v22. A chi-square test was used to identify the correlation between molecular grade and clinical parameters. Survival curves were evaluated by the Kaplan–Meier (KM) method, and the log-rank test was done to compare survival distribution between groups. All statistical tests performed were two-sided, with p-values less than 0.05 as the threshold for significance.

## Results

### Clinical Characteristics of Study Cohort

A summary of the clinical characteristics of this study cohort is shown in **Table 1**. A total of 1,275 adult diffuse gliomas were recruited in this study. The male-to-female ratio was 1.4:1. The mean age and median age at diagnosis were 45.6 and 45 years, respectively. The median and mean follow-ups were 3.9 and 6.7 years, respectively. Nearly 65% of the patients underwent gross total resection. Radiotherapy was given in >50% of patients, and chemotherapy was given to nearly half of the patients. In China, healthcare is not strictly nationalized according to districts unlike other countries, and because of our neurosurgical expertise in lower-grade gliomas, we had more referrals for this group (43.7% Grade II, 23.8% Grade III). Univariate analyses showed that histological grades, age, the extent of resection, chemotherapy, and radiotherapy were significantly associated with OS (**Supplementary Figure 1**). The complete data of 1,275 cases can be found in https://www.surgery.cuhk.edu.hk/btc/hsbc/.

We stratified the adult gliomas into 6 molecular grades based on IDH mutations, 1p19q codeletion, TERTp mutation, BRAF mutation, EGFR amplification, 10q loss, and H3 mutations. The molecular grading scheme was as follows:

Molecular Group 1: IDH mutant, 1p19q codeleted
Molecular Group 2: IDH mutant, 1p19q non-codeleted, TERT mutant
Molecular Group 3: IDH mutant, 1p19q non-codeleted, TERT wild type
Molecular Group 4: IDH wild type, BRAF mutant
Molecular Group 5: IDH wild type, BRAF wild type, with no evidence showing TERTp mutation, EGFR amplification, 10q loss, and H3 mutation
Molecular Group 6: IDH wild type, positive for TERTp mutation, EGFR amplification, 10q loss, or H3 mutations

The numbers of different tests carried out on this cohort are shown in **Supplementary Table 2**.

Overall, there were 270 cases of molecular Group 1, 59 cases of molecular grade 2, 248 molecular grade 3, 27 cases of molecular grade 4, 117 cases of molecular Group 5, and 307 cases of molecular grade 6 (**Table 1**). A total of 247 cases were unclassifiable for the six molecular groups, all due to a lack of data for some biomarkers. We found that molecular grading was significantly associated with histological grade (p < 0.001). Molecular Group 1 was enriched for WHO Grade II tumors and absent in WHO Grade IV tumors. Nearly 80% (76.3%) of molecular Group 1 tumors were of WHO grade II. Molecular Group 6 was highly enriched for WHO Grade IV, and nearly 70% of molecular grade 6 tumors were of WHO grade IV. Molecular groups were not associated with sex. The 27 cases of molecularly Group 4 were not epithelioid, PXA-like, or gangliogliomas on histological review.

**Figure 1:**
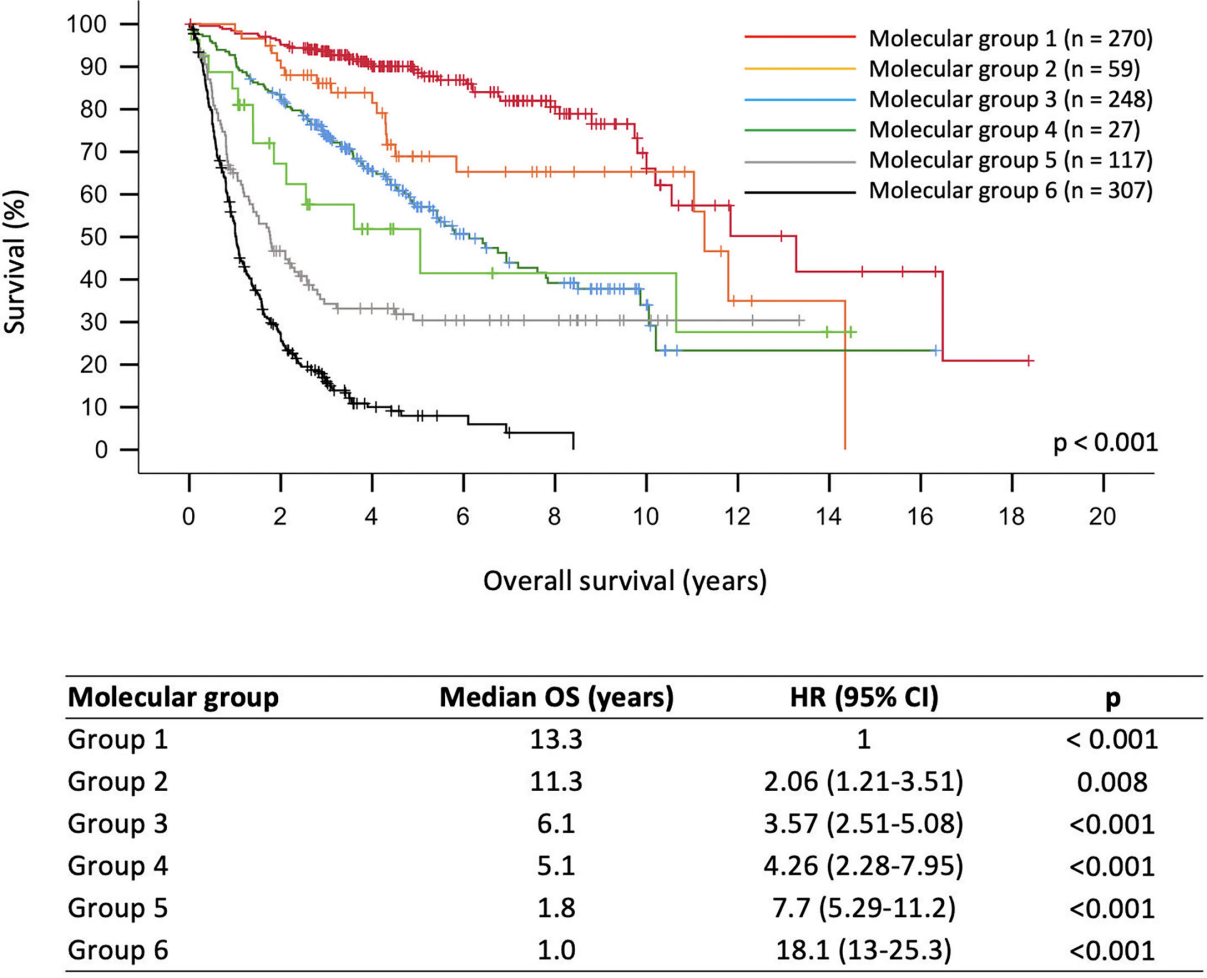
Overall survival curves of adult gliomas with respect to molecular Group.

**Figure 2:**
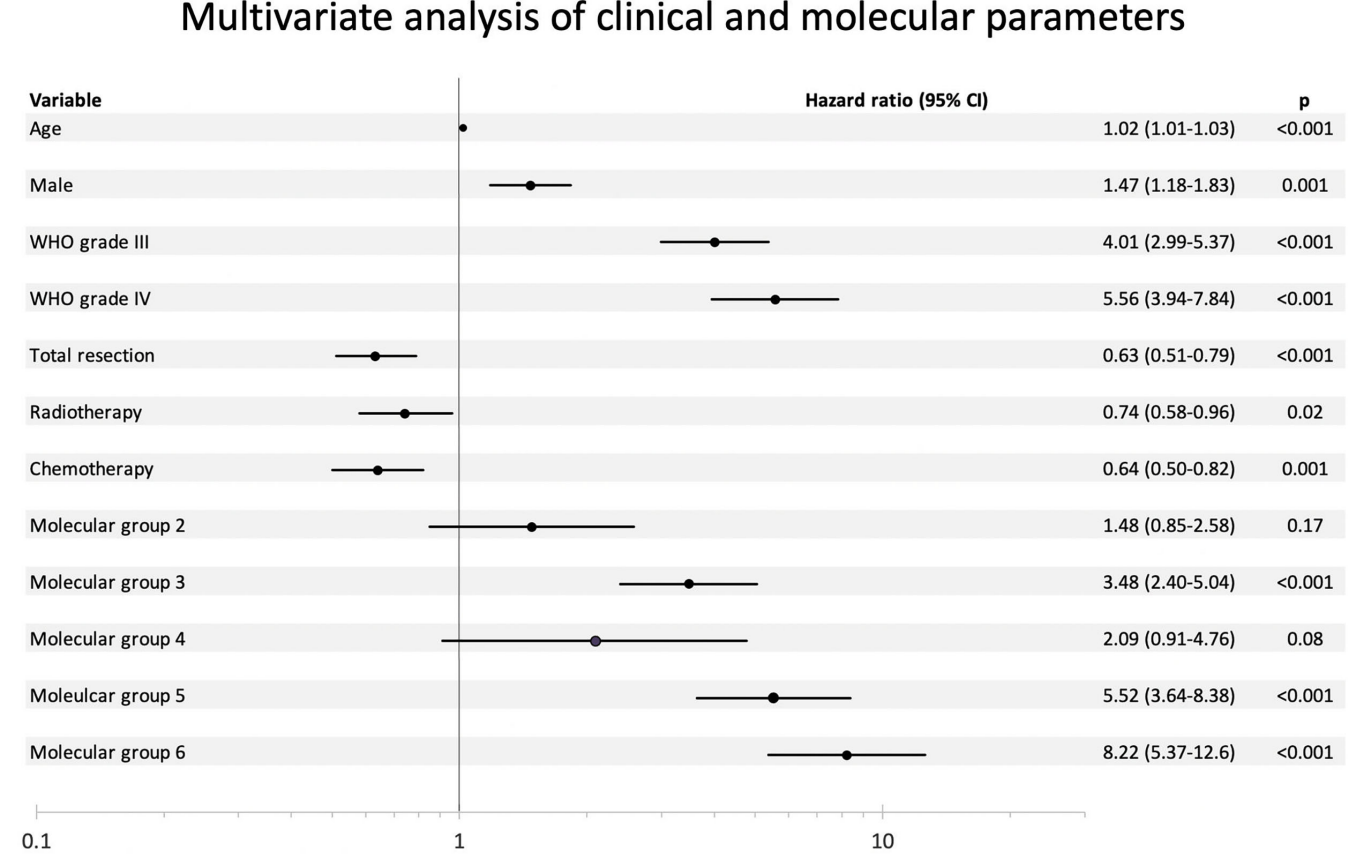
Multivariate analysis of clinical and molecular parameters.

As shown in **Figure 1**, molecular grades defined by molecular signatures were strongly associated with prognostication across the whole cohort (p < 0.001). Univariate analyses showed that all molecular grades were significantly associated with OS (**Supplementary Figure 1** and **Figure 1**). Multivariable analysis showed that molecular groups had independent prognostic value (p < 0.001) across the cohort by adjusting for age, gender, histological grades, tumor resection, radiotherapy, and chemotherapy. Notably, molecular Groups 3, 5, and 6 tumors demonstrated significantly worse prognosis, and molecular Groups 2 and 4 tumors showed trends of unfavorable prognosis as compared to the reference group (molecular Group 1) (**Figure 2**).

**Figure 3:**
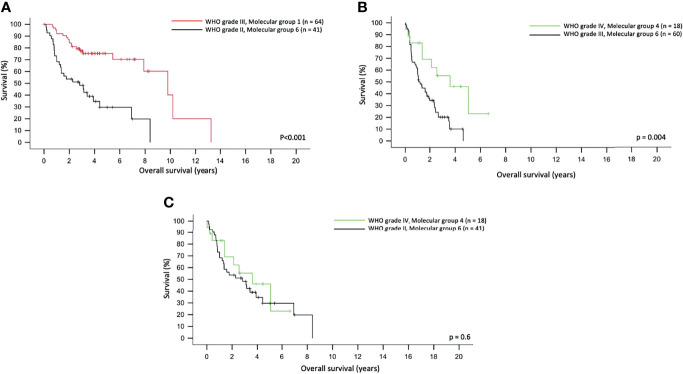
Molecular group superseded the histological grading in predicting overall survival in adult gliomas **(A)**. WHO grade III, molecular Group 1 tumors had a longer overall survival as compared to WHO grade II, molecular Group 2 tumors. **(B)** WHO grade III, molecular Group 6 tumors had a significantly shorter overall survival as compared to WHO grade IV, molecular Group 4 tumors. **(C)** WHO grade IV, molecular Group 4 and WHO grade II, molecular Group 6 tumors did not differ in overall survival.

We also tried to compare the power of prognostication of the molecular groups against histological grades in different scenarios. In three scenarios, the molecular groups were better than histological grades in prognostication. **Figure 3A** shows that WHO Grade III molecular Group 1 tumor demonstrated significantly longer OS than WHO Grade II molecular Group 6 tumors (p < 0.001). **Figure 3B** also illustrates that WHO Grade IV, molecular Group 4 tumors in fact had a remarkably longer OS as compared to the WHO Grade III, molecular Group 6 tumors (p = 0.004). Interestingly, WHO Grade IV molecular Group 4 tumors had similar OS as compared to the WHO Grade II molecular Group 6 tumors (**Figure 3C**). A total of 93 cases of IDH mutant, 1p19q non-codeleted gliomas were evaluated for CDKN2A; 11.8% of cases (11/93) were positive for CDKN2A homozygous deletion, and this was not associated with OS (p = 0.235).

We next retrieved an unselected adult glioma cohort with relevant clinical and molecular data from TCGA (10). The proportion of available biomarkers relevant to this study in this TCGA validation cohort is listed in **Supplementary Table 3**. When the molecular grading scheme of this study was used in TCGA cohort, there were 169 cases of Group 1, 8 cases of Group 2, 146 cases of Group 3, 7 cases of Group 4, 140 cases of Group 5, and 303 cases of Group 6. When tested against OS, the six molecular groups remain statistically significant (p < 0.001) (**Supplementary Figure 2**).

## Discussion

The diagnostic protocols for brain tumors have been vastly changed in the advanced centers due to the advent of next-generation sequencing, for both DNA and RNA and also genome-wide methylation profiling. The former can evaluate gene mutations or gene fusion on a panel of a large number of genes, while the latter can map all brain tumors according to their methylation profiling into 91 methylation classes (7). Copy number variations (CNVs) can be obtained from both methods. Both have been widely used in the day-to-day fine diagnosis of brain tumors as well as research (8, 30, 38, 39). The WHO 2016 Classification of CNS Tumors for the first time introduced molecular criteria in the diagnosis of some brain tumors, and many centers in the world use an integrated, multi-layered approach in the pathological diagnoses of brain tumors, integrating histological features, WHO grades, and molecular characteristics. Adult diffuse astrocytic tumors are classified into IDH-mutant and IDH wild type, and oligodendrogliomas are defined by both IDH mutation and 1p19q codeletion by the WHO 2016 Classification. However, grading of adult diffuse gliomas remains entirely histological depending mainly on mitoses, cellular anaplasia, necrosis, and microvascular proliferation.

Most general pathology laboratories in the world are not in a position to carry out next-generation sequencing or genome-wide methylation profiling routinely on all their neurosurgical specimens, as these tests remain expensive and demanding on expertise for interpretation, e.g., t-distributed stochastic neighbor embedding (t-SNE) plots. Most national types of health finance systems are unable to support such extensive genomic tests regularly. However, most pathology departments in the more advanced societies will have the capacity for single-gene studies and molecular tests like Sanger sequencing, for example, for BRAF V600E, IDH1 R132, and IDH2 R172H. Similarly, FISH is also widely available in pathology departments, especially in the context of Her-2 testing for breast cancers. We therefore believe that there is a need to develop a relatively simple molecular grading algorithm for the gliomas based on single-gene testing based on current literature on the molecular pathology of gliomas (15, 32, 33, 40–42). There are many excellent studies on the grading of special groups of gliomas by a panel of single genes. For example, TCGA concluded that IDH and 1p19q status satisfactorily grades diffuse lower-grade gliomas, while Eckel-Passow et al. further fine-graded lower-grade gliomas with the addition of TERT status (14, 15).

Mutations of the promoter region of TERT have been suggested to be a key terminal event in the evolution of glioblastomas, but it has an intriguing property of being mutated also in oligodendrogliomas, which are mostly low-grade gliomas (43). The reason for its dual and seemingly contradictory properties is unknown. The dual impact of TERT can be seen in that other than being present in IDH mutant, 1p19q codeleted tumors, it was also a prognosticating factor among IDH mutant 1p19q non-deleted tumors (44, 45) and, in this series, the difference between molecular grade 2 (TERT mutant, IDH mutant, 1p19q non-codeleted) and molecular grade 3 (TERT wild type, IDH mutant, 1p19q non-codeleted). However, molecular group 2 remains a small group in our cohort (n = 59).

Other than lower-grade gliomas, there have also been a few studies in the literature devoted to molecular-grade special groups of gliomas by single-gene studies (32, 33), but we are only aware of one previous attempt to use a panel of single-gene status to grade holistically all adult gliomas (46). In this study, Jiao et al. showed that gliomas can be satisfactorily graded for prognosis by complete sequencing of IDH1/2, ATRX, CIC, and FUBP1. However, the latter genes are large, and sequencing of entire genes is laborious and expensive.

The biomarkers used in this study were known in the literature on their own. Group 1, IDH mutant, 1p19q codeleted is the regular oligodendroglioma as currently defined by WHO Classification, and Group 6 constitutes the bulk of the regular IDH wild-type glioblastomas as defined by cIMPACT-NOW update 3 (47). Group 5 would constitute most of the IDH wild-type lower-grade gliomas that do not fulfill the molecular criteria of glioblastoma (33, 48, 49). It is also interesting that in our molecular Group 4, BRAF V600E was able to impart a better prognosis among the IDH wild-type gliomas. This is consistent with the few papers concerning the potentially better prognosis of BRAF V600E in adult gliomas (16–18). The overall number in this IDH wild type, BRAF mutant group is small (27/1,028 cases), consistent with the overall low percentage of BRAF V600E in adult gliomas (50). These tumors were not epithelioid, PXA-like, or gangliogliomas on histological review; similarly in the three references quoted, there was also no mention of such histological features.

For glioblastomas, cIMPACT and the new WHO (2021) Classification recommended the use of EGFR amplification, TERT, and chromosome 7+/10− as criteria for diagnosis of molecular glioblastoma in IDH wild-type adult gliomas, which do not show diagnostic histological features like necrosis and microvascular proliferation (1, 47). For IDH mutant histologically lower-grade gliomas, the molecular diagnostic criterion for glioblastoma is the homozygous deletion of CDKN2A/B (51). In this study, we have used extensively EGFR amplification and 10q loss in addition to TERT, as 10q loss has been shown to be associated with glioblastomas for many years (52, 53). The WHO (2021) and cIMPACT criteria actually recommend combined whole chromosome 7 gain and 10 losses (1, 47), with molecular events that are usually not tested in a general pathology laboratory, and the critical reference for this recommendation used methylation profiling to test for whole chromosomes (7).

The molecular grading scheme we propose can easily be made available in general pathology departments. Adoption of this system provides a fine prognostication for gliomas and in, some instances, is superior to the histological grading, as shown in **Figure 3**. The use of this scheme also fulfills many of the recommended markers of the WHO Classification. While most of the biomarkers used in this grading scheme including 1p19q codeletion, IDH2 mutations, 10q loss, and TERT mutation do not have any immunohistochemical tests, IDH1 and BRAF V600E mutations are readily tested by immunohistochemistry, and these can be used in busy diagnostic laboratories. We recognize that not all single-gene biomarkers that were described in the new WHO Classification and cIMPACT (e.g., CDKN2A/B) have been included in the grading scheme. In our cohort of molecular Grade 2 (IDH mutant, 1p19q non-codeleted, TERT mutant) and Grade 3 (IDH mutant, 1p19q non-codeleted, TERT wild type) tumors, we were unable to demonstrate a statistical significance between cases with or without homozygous CDKN2A deletion. Marker and Pearce also showed that CDKN2A deletion was not associated with prognostication in Grades II and III IDH-mutant astrocytomas (54). Methylated MGMT has been known for a long time to be a favorable prognostic marker in glioblastomas (55) and predicts their response to alkylating chemotherapy (56, 57). However, it is not in regular use for the lower-grade glioma as a prognosticator, and most of the low histological grade IDH mutant gliomas are likely to be MGMT methylated.

We also recognize the limitations of our study, as we used retrospective analyses of existing data, and it would be impossible for us to re-do a new panel of single-gene markers for 1,275 cases according to the recommendation of the new WHO Classification. Ours is not a proposal to replace the new WHO Classification (1) but merely a proof of principle that a set of well-placed single-gene biomarkers can fully cover risk stratification in adult gliomas. Even in the new WHO Classification (1), there are cases that do not fit and are classified as NEC (Not Elsewhere Classified), e.g., IDH wt gliomas that do not exhibit EGFR amplification or TERTp or combined whole chromosomes +7/−10q. It is possible that other algorithms may have developed that use some biomarkers might be shown to be equally useful in molecular grading of gliomas in the future. But our study showed that a simple molecular grading scheme such as the one proposed can satisfactorily fine-grade adult gliomas, and these tests can be performed relatively quickly and are highly achievable in practically all pathology laboratories. This grading scheme will provide critical and timely information for immediate clinical management of glioma patients.

## Data Availability Statement

The datasets presented in this study can be found in online repositories. The names of the repository/repositories and accession number(s) can be found in the article/**Supplementary Material**.

## Ethics Statement

The studies involving human participants were reviewed and approved by The Joint Chinese University of Hong Kong–New Territories East Cluster Clinical Research Ethics Committee; the Ethics Committees of Huashan Hospital, Shanghai; and The First Affiliated Hospital of Zhengzhou University, Zhengzhou. The patients/participants provided their written informed consent to participate in this study.

## Author Contributions

The authors confirm contribution to the paper, as follows: study conception and design: AC, W-SP, HL, and H-KN. Data acquisition: Z-FS, KL, W-WW, HC, NC, DC, X-ZL, Z-YZ, and YM. Analysis and interpretation of data: AC, Z-FS, KL, NC, and H-KN. Drafting of the manuscript: AC and H-KN. All authors listed have made a substantial, direct, and intellectual contribution to the work and approved it for publication.

## Funding

This study was supported by the Health and Medical Research Fund (HMRF), the Food and Health Bureau of Hong Kong (07180736); the National Natural Science Foundation of China (82072020, 81702465, U1904148 and U1804172); and Shanghai Municipal Science and Technology Major Project (2018SHZDZX01).

## Conflict of Interest

The authors declare that the research was conducted in the absence of any commercial or financial relationships that could be construed as a potential conflict of interest.

## Publisher’s Note

All claims expressed in this article are solely those of the authors and do not necessarily represent those of their affiliated organizations, or those of the publisher, the editors and the reviewers. Any product that may be evaluated in this article, or claim that may be made by its manufacturer, is not guaranteed or endorsed by the publisher.
