# Supplementary material for: Combinations of Single-Gene Biomarkers Can Precisely Stratify 1,028 Adult Gliomas for Prognostication
Source: Front Oncol. 2022 Apr 26;12:839302. doi: 10.3389/fonc.2022.839302 (PMC9090434; doi:10.3389/fonc.2022.839302)
Supplement: Supplementary file 1 [file DataSheet_1.pdf]

Supplementary Table 1: Primers used in this study

Gene	Forward primer sequence	Reverse primer sequence	Annealing temperature (°C)
BRAF	5′-TGCTTGCTCTGATAGGAAAATG-3′	5′-CCACAAAATGGATCCAGACA-3′	62
H3.3	5′-CATGGCTCGTACAAAGCAGA-3′	5′-CAAGAGAGACTTTGTCCCATTTTT-3′	62
H3.1	5′-TTTCCTTTCCTCCACAGACG-3′	5′-CGGTAACGGTGAGGCTTTT-3′	62
IDH1	5’-CGGTCTTCAGAGAAGCCATT-3’	5’-CACATTATTGCCAACATGAC-3’	62
IDH2	5’-AGCCCATCATCTGCAAAAAC-3’	5’-CTAGGCGAGGAGCTCCAGT-3’	62
TERT promoter	5’-GTCCTGCCCCTTCACCTT-3’	5’-CAGCGCTGCCTGAAACTC-3'	68

Supplementary Table 2. Numbers of molecular tests carried out in this study

		N	Number of case done
IDH			1275
	mutant	649	
	wild-type	626	
1p19q			1012
	codeleted	272	
	non-codeleted	740	
TERT			1234
	mutant	516	
	wild-type	718	
BRAF			407
	mutant	31	
	wild-type	376	
EGFR			537
	amplified	76	
	non-amplified	461	
H3.3			308
	mutant	27	
	wild-type	281	
10q			149
	deleted	57	
	non-deleted	92	

Supplementary Table 3. Numbers of molecular tests carried out in TCGA study			
		N	Number of cases done
IDH1/2			
	mutant	532	
	wild-type	455	987
1p19q			
	codeleted	169	
	non-codeleted	922	1091
TERTp			
	mutant	161	
	wild-type	167	328
BRAF			
	mutant	8	
	wild-type	793	801
EGFR			
	amplified	292	
	non-amplified	796	1088
H3.1 and H3.3			
	mutant	2	
	wild-type	799	801
10q			
	deleted	61	
	non-deleted	1027	1088

Supplementary Figure 1

Univariate analysis of clinical and molecular variables

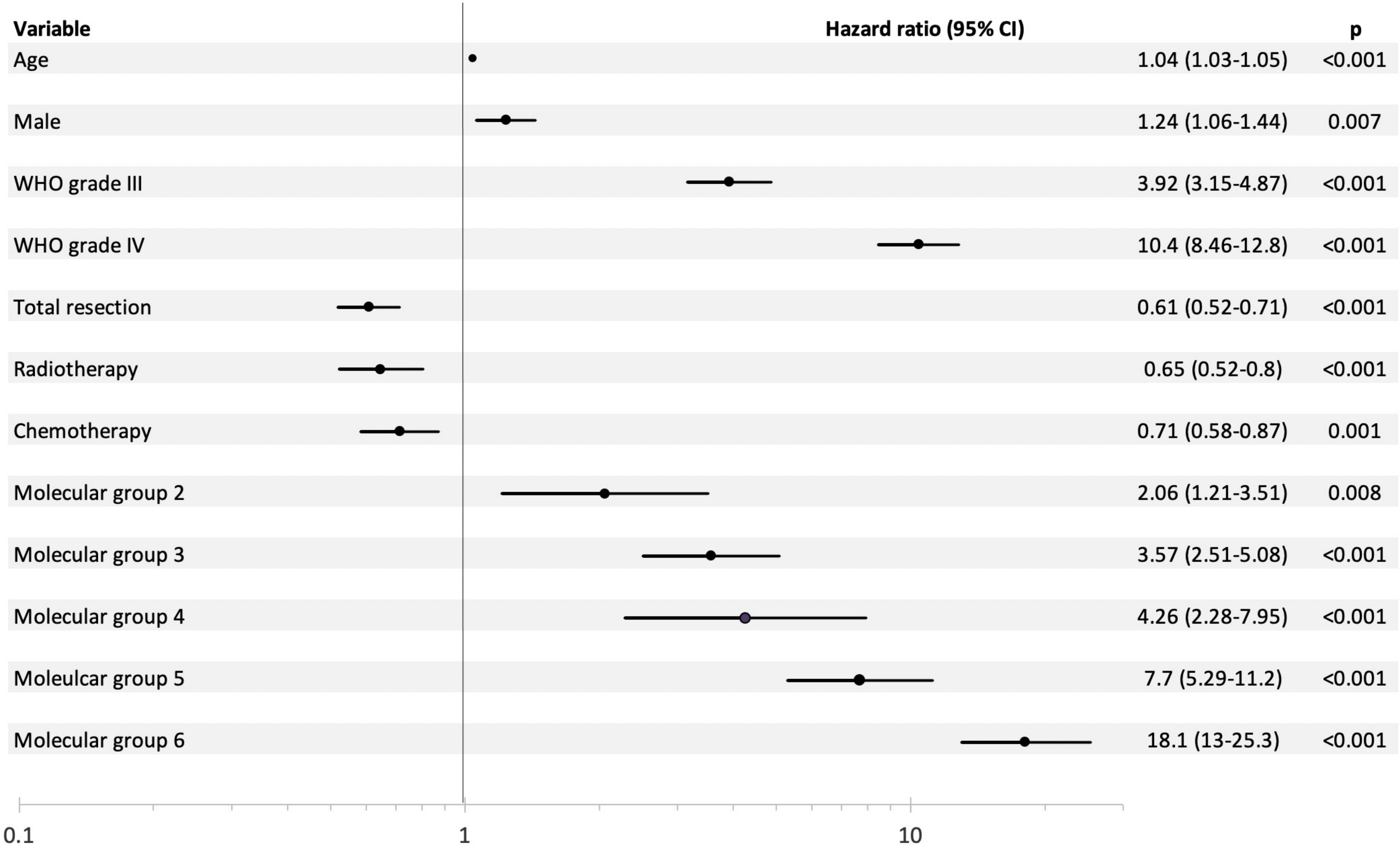

Supplementary Figure 2

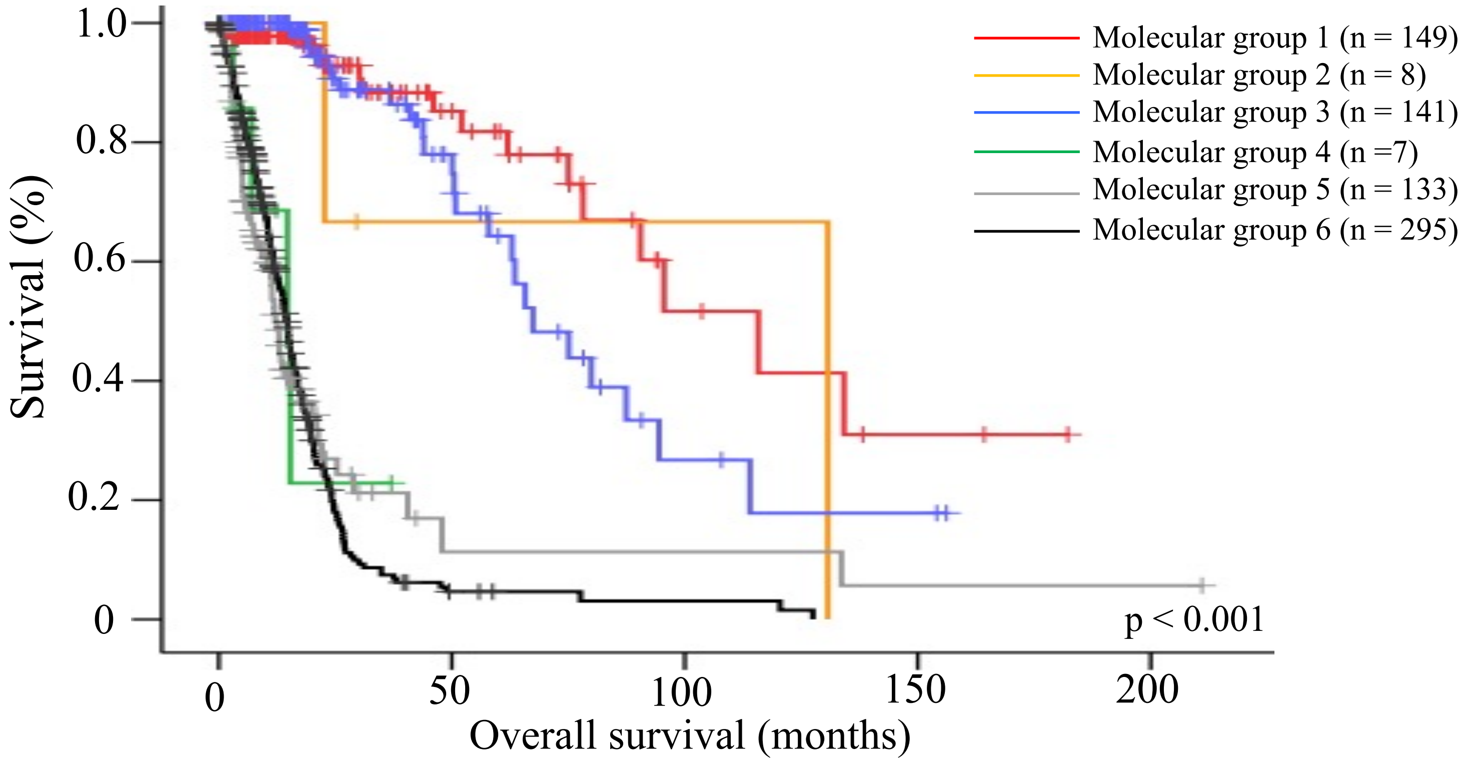

**Molecular grade**	**Median OS (years)**	**HR (95% CI)**	**p**
Group 1	9.6	1	< 0.001
Group 2	10.9	2.19 (0.51-9.52)	0.295
Group 3	5.6	1.49 (0.80-2.79)	0.206
Group 4	1.2	10.57 (3.53-31.60)	<0.001
Group 5	1.0	11.82 (6.92-20.21)	<0.001
Group 6	1.2	12.79 (7.72-21.18)	<0.001
